# Comparing the yield of oropharyngeal swabs and sputum for detection of 11 common pathogens in hospitalized children with lower respiratory tract infection

**DOI:** 10.1186/s12985-019-1177-x

**Published:** 2019-06-24

**Authors:** Le Wang, Shuo Yang, Xiaotong Yan, Teng Liu, Zhishan Feng, Guixia Li

**Affiliations:** 1 Institute of Pediatric Research, Children’s Hospital of Hebei Province, Shijiazhuang, 050031 China; 2 Hebei General Hospital, Shijiazhuang, 050000 China

**Keywords:** Oropharyngeal swabs, Sputum, Children, LRTI

## Abstract

**Background:**

Advances in molecular laboratory techniques are changing the prospects for the diagnosis of viral infectious diseases. Multiplex polymerase chain reaction assay (multiplex-PCR) can detect dozens of pathogens simultaneously, greatly reducing turnaround time (TAT) and improving detection sensitivity. But as a double-edged sword, due to the high sensitivity of PCR, the type of respiratory specimens is critical to diagnosis. In this work, we performed a head-to-head comparison to evaluate the multiplex-PCR yields between two samples, sputum and flocked oropharyngeal swabs (OPS).

**Methods:**

Eleven common respiratory pathogens were tested in hospitalized children< 13 years of age who met the criteria for lower respiratory tract infection by GeXP-based multiplex-PCR of paired OPS and sputum.

**Results:**

From January to June 2018, 440 children with paired OPS and sputum were tested. The positive rate was 84% (369/440) for OPS and 88% (386/440) for sputum (*p* = .007). The frequency of detection of HRV, RSV, Influenza A virus, HMPV, parainfluenza virus, adenovirus, *M. pneumoniae,* coronavirus, bocavirus and *C. pneumoniae* in sputa was higher than that of OPSs (all *p* < .001). Both types of specimens had similarly very good kappa values for most of pathogens, except for *Mycoplasma pneumonia* (κ = 0.61) and *Chlamydia pneumoniae* (κ = 0.24)*.* Additionally, 79.3% (349/440) of cases showed consistent results between the two types of samples, and they were significantly younger than patients with inconsistent results (*p* = .002).

**Conclusions:**

Flocked oropharyngeal swabs and sputum performed similarly for the detection of common respiratory pathogens in hospitalized children by multiplex-PCR, except for *Mycoplasma pneumoniae* and *Chlamydia pneumoniae*. Young patients are likely to have consistent results between the two specimens.

**Electronic supplementary material:**

The online version of this article (10.1186/s12985-019-1177-x) contains supplementary material, which is available to authorized users.

## Background

The lower respiratory tract infection (LRTI) is one of the most common infections in the world, leading to significant morbidity and mortality in children [1]. Accurate and early etiologic diagnosis will help clinicians to initiate appropriate antimicrobial therapy [2]. Although molecular techniques directly applied to respiratory tract specimens could detect multiple pathogens with high specificity and sensitivity [3], the choice of sample type and sampling method is critical for optimal diagnostic efficacy [4].

Currently, specimens for diagnostic purpose by PCR include oropharyngeal (OP) swabs, nasopharyngeal (NP) swabs, NP aspirates, OP suction and sputum. Although upper respiratory tract specimens are commonly used in children with respiratory viral and some bacterial infections, there is concern whether the results reflect the cause of lower respiratory tract infection [5]. Lots of studies have compared the yields of these upper respiratory tract specimens, and by PCR to identify viral or bacterial infections by PCR, they have found that the sensitivity of aspirate (or suction) is greater than that of swabs [6–8]. Compared with specimen from upper airway, excellent diagnostic sensitivity is observed when sputum is available [4, 9–11]. However, for children, especially young patients who cannot expectorate, a sterile negative pressure suction catheter is applied to obtain OP suction. Young children and parents may find this relatively invasive and distressing procedure unacceptable, thus limiting its use in routine clinical practice [9, 12]. In addition, those oropharyngeal suction or sputa, presumably from, or contaminated by secretions from the upper respiratory tract [13, 14]. Therefore, it is important to assess the prevalence of pathogens in different types of specimens.

To the best of our knowledge, there have been no reports describing the adequacy of different types of specimen for the simultaneous detection of several viruses and atypical bacteria using multiplex-PCR. Therefore, we used multiplex-PCR to compare the detection of 9 types of viruses and 2 atypical bacteria in paired sputum and OPS samples from children with lower respiratory tract infection.

## Subjects and methods

### Study population

During the first period of study (from January to December of 2017), in order to evaluate the clinical application of multiplex-PCR method, we enrolled LRTI children from three hospital (The 2th Affiliated Hospital of WNU, Children’s Hospital of Hebei Province and The 1st Hospital of Shijiazhuang), the sputum samples were collected. During the second period, to compare the detection rates between sputum and OPS samples, children with LRTI was enrolled from January to June of 2018 at Children’s hospital Hebei Province. Both sputum and OPS samples were collected. All patients were children diagnosed with LRTIs (including pneumonia, bronchitis and bronchiolitis). LRTIs were diagnosed based on the clinical and radiologic findings. Enrollment of children in the study followed the diagnosis of dominant symptoms of an acute or worsening cough or a clinical presentation. The age ranged from 1 day to 13 years. Detailed demographic and clinical information were collected from the inpatient electronic medical records system. After conducting a pilot study to examine feasibility, a trained research assistant approached consecutive patients with LRTI and collected clinical and demographic data from guardians.

### Sample collection

Samples will be excluded if the sputum and OPS were not collected in the same day. Pathogens were detected using different sampling methods including flocked oropharyngeal swabs (OPSs) and sputum. Oropharyngeal swabs were collected using a commercially available nylon flocked swab (Mrk Tech., Shenzhen, China). The swab was gently rolled on the tonsils and the posterior wall of the oropharynx with enough pressure and ensure not be contaminated by the normal flora of the mouth. Then the swab was placed in 2 mL VTM (Hopebio Technologies, Qingdao, China).

Patients were asked to cough, and the expectorated sputum was collected. If the child is too young to cough, a sterile negative pressure suction catheter is applied to obtain the oropharyngeal suction into transport tube containing VTM. The sample was stored at 4 °C for the same day pathogen nucleic acid extraction.

### Multiplex-PCR

For OPS sample, place the VTM on a vortex for 10 s to wash the virus and virus-containing cells on the OPS. For sputum sample, thoroughly mix the sputum sample with VTM and aspirate 200 μL supernatant for the following nucleic acid extraction. The 3 μL internal control was added into every extracted sample.

Nucleic acids were extracted using the nucleic acid extraction kit on an automated extraction workstation (Smart LabAssist-16/32) according to manufacturer’s instructions (Health Gene Technologies, Ningbo, China).

For multiplex reverse transcription (RT), the mix of all reverse primers (RT primer) from target pathogens (Additional file 1: Table S1) was used. RT was performed in a total volume of 20 μL containing 5 μL of nucleic acid sample, 14 μL Premix, 1 μL (40 units) RT-PCR reverse transcriptase. RT was carried out as follows, 25 °C for 5 min; and then 50 °C for 15 min. The reaction was terminated by incubation at 95 °C for 2 min. Multiplex PCR was performed in following steps: step 1, 94 °C for 30s, 65 °C to 60 °C touchdown PCR for 30s and 72 °C for 1 min, repeated for 6 cycles; step 2, 94 °C for 30s; 60 °C for 30s; 72 °C for 1 min, repeated for 29 cycles; step 3, 72 °C for 10 min, step 4, 4 °C. The 10 μL amplified products were added into the 287 μL loading buffer and 3 μL SizeStandard-400, and then assessed using the GenomeLab GeXP Genetic Analysis System (Beckman Coulter).

Pathogens detection was performed using the Respiratory Pathogens Multiplex Kit (Health Gene Tech., Ningbo, China), which is a multiplex PCR-capillary electrophoresis fragment analysis designed to detect respiratory microbes including Influenza A (Flu A), Influenza B (Flu B), human parainfluenza virus (HPIV), respiratory syncytial virus (RSV), rhinovirus (HRV), adenovirus (ADV), human metapneumovirus (HMPV), human bocavirus (HBoV), human coronavirus (HCoV), *Chlamydia pneumoniae* (Cp) and *Mycoplasma pneumoniae* (Mp). The analysis was then performed in an automated manner following the established protocol and the data were compiled by the GeXP system software provided by Beckman Coulter.

### Statistical analyses

The overall positive rates of pathogens between OP swabs and sputum samples were compared using McNemar’s test. The detection yields of any microbes between two specimens were compared using the χ^2^ or Fisher’s exact test. Agreement of the results between OP swabs and sputum specimens was assessed using Kappa statistics (κ value 0.21–0.4 fair, 0.41–0.6 moderate, 0.61–0.8 substantial and 0.81–1 almost perfect) [15]. Analyses were performed using SPSS software version 19.0 (SPSS Inc., Chicago, USA) and GraphPad Prism version 6.00 (GraphPad, La Jolla, California). Statistical significance was concluded if *P* < .05.

### Ethics consideration

The study was approved by the Children’s hospital Hebei Province Ethics Committee (number 2017016). The legal guardian(s) or parent(s) of the children provided written informed consent for sample collection and clinical record review.

## Results

### Study population

During the first stage, in order to evaluate the clinical application of our multiplex-PCR method, a total of 1650 sputum specimens, collected from three hospitals, were subjected to mono-RT-PCR of 11 pathogens and 18,150 single-sequencing verifications were performed. Compared to direct sequencing, the multiplex-PCR method showed 100% sensitivity (positive coincidence rate), 99.94% specificity (negative coincidence rate) and 99.95% accuracy (total coincidence), kappa value = 0.997 (*p* < 0.01).

**Fig. 1:**
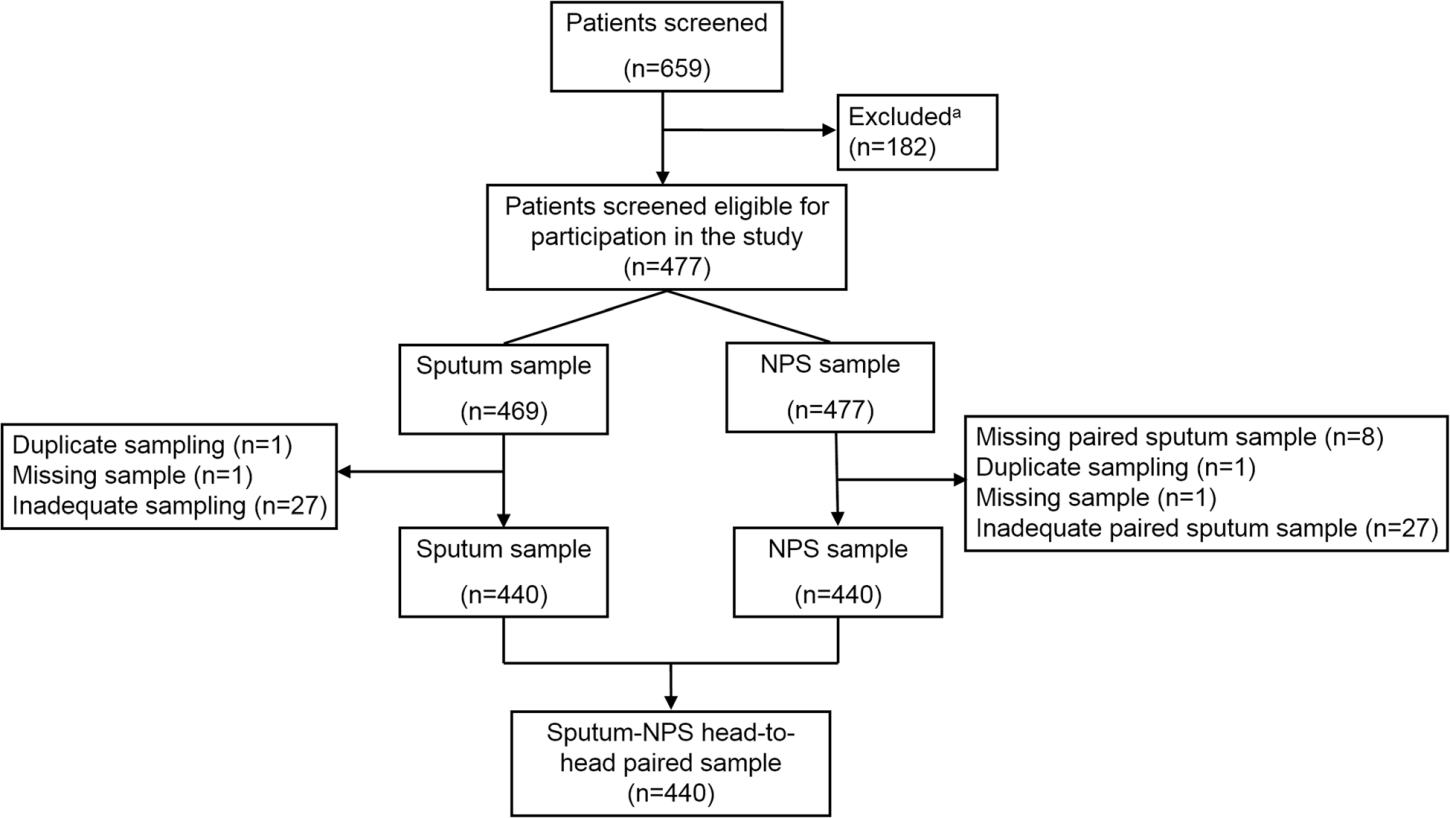
Flow-chart of patient enrollment. ^a^: samples from 8 cases were not collected by the nurses who have received a sampling training. One hundred five patients refused to provide the paired OPS specimens. Sixty-nine sputum and OPS were not collected in the same day. Abbreviation: HRV, human rhinovirus; RSV, respiratory syncytial virus; InfA, influenza A; HMPV, human metapneumovirus; HPIV, human parainfluenza virus; InfB, Influenza B; ADV, adenovirus; MP, *Mycoplasma pneumoniae*; HCoV, human coronavirus; HBoV, human bocavirus; CP, *Chlamydia pneumoniae*

During the second stage, to compare the detection rates between sputum and OPS samples, a total of 659 hospitalized children< 13 years were enrolled from January to June of 2018. Among them, 182 were excluded because: i) 8 samples were not collected by the nurses who have received a sampling training; ii) 105 patients refused to provide the pairing OPS specimens; iii) 69 sputum and OPS were not collected on the same day. We also excluded samples taken from the same patients, which were collected less than a week apart, and a few samples that were inadequate to conduct all assays. The flow chart for including and excluding participants is shown in Fig. 1. Finally, paired OPS and sputum samples were collected from 440 patients, and the main diagnosis was pneumonia (63.9%, Table 1). Participants were more likely to be male (269; 61.1%) with a median age of 0.7 years (interquartile range 0.3–1.2).

**Table 1:** Number of LRTI children enrolled by diagnosis

Diagnosis	Number	Percentage
Lobar pneumonia	281	63.9
Bronchial pneumonia	97	14.9
Bronchitis/capillary bronchitis	62	10.7

### Sputum and OPS concordance on certain pathogens

Overall, the positive detection rate of OPS was 84% (369/440), and sputum was 88% (386/440). For most pathogens, sputum specimens showed a significantly higher positive rate than OPS (Table 2, *P* = 0.007, Table 3, all *P* < 0.001). The only virus for which this was not the case was influenza B (91% for OPS, 89% for sputum, Table 3).

**Table 2:** Comparison of multiplex-PCR Results between Sputum and OPSs Samples

		No. (%) of sputum result	
		Positive	Negative
No. (%) of OPSs result	Positive	360 (81.8)	26 (5.9)
	Negative	9 (2.0)	45 (10.2)

*P* = 0.007 by McNemar’s test

**Table 3:** Detection of 11 types of pathogens according to specimen type

Pathogens	No. of positive sputum and/or OPS^a^	Sputum (%)	OPS (%)	*P* value	*kappa* value
HRV	137	131 (96)	114 (83)	<.001	0.836
RSV	124	121 (98)	111 (92)	<.001	0.906
Influenza A	74	73 (99)	68 (92)	<.001	0.941
HMPV	72	69 (96)	65 (90)	<.001	0.912
HPIV	34	33 (97)	28 (82)	<.001	0.877
Influenza B	35	31 (89)	32 (91)	<.001	0.880
Adenovirus	23	20 (87)	19 (83)	<.001	0.812
*M. pneumoniae*	20	20 (100)	9 (45)	<.001	0.610
HCoV	17	15 (88)	14 (82)	<.001	0.822
HBoV	14	14 (100)	12 (86)	<.001	0.921
*C. pneumoniae*	14	12 (86)	4 (29)	<.001	0.240

^a^Column total adds to more than the number of ARIs with any pathogen present, because children with more than 1 pathogen identified are also recorded

*HRV* indicates human rhinovirus, *RSV* respiratory syncytial virus, *HMPV* human metapneumovirus, *HPIV* human parainfluenza virus, *HCoV* human coronavirus, *HBoV* human bocavirus

For most organisms, the consistency between OPS and sputum specimens was almost perfect (κ value 0.81–1). However, for certain organisms, including *M. pneumoniae* and *C. pneumoniae*, sputum specimens appear to be superior to OPS specimens, as the κ values were 0.61 and 0.24. respectively (Table 3).

### Sputum and OPS concordance on cases

The multiplex-PCR results for 349 (79%) paired specimens were consistent (Table 4). By either collection assay, there was at least one pathogen detected in 304 cases (69.1%): 230 with 1 pathogen and 74 with 2 or 3 pathogens. The left 45 (10.2%) LRTIs with no microbes were identified in either specimen. RSV was the most common single virus found in concordant specimens (17%). Of the 91 (21%) discordant specimen pairs, completely inconsistent results were identified in 2 (0.5%) and partially consistent in 89 (20%). Among the partially consistent cases, more types of organisms in sputum were identified in 71 cases (16.1%), more types in OPS in 16 cases (3.6%) (Table 4).

**Table 4:** Sputum and OPS Concordance

Items			Number	Percentage
Concordant Sputum/OPS paired specimens			349	79.3%
No pathogen identified			45	10.2%
Single pathogen identified			230	52.3%
RSV			75	17.0%
HRV			48	10.9%
HMPV			40	9.1%
Influenza A			33	7.5%
Influenza B			15	3.4%
HPIV			8	1.8%
MP			5	1.1%
HBoV			3	0.7%
HCoV			3	0.7%
Two or three pathogens identified			74	16.8%
Discordant Sputum/OPS paired specimens			91	20.7%
Completely inconsistent			2	0.5%
Partially consistent: more types of pathogen in Sputum			71	16.1%
Partially consistent: more types of pathogen in OPS			16	3.6%
Partially consistent: 1 pathogen inconsistent, others are consistent			2	0.5%

Children with consistent results of sputum and OPSs were significantly younger than those with inconsistent results (median age 0.6 vs. 1.0 years, *p* = 0.002). But this difference was not observed in the gender distribution (Table 5).

**Table 5:** Age and gender characteristics of concordant and discordant cases

	Sputum/OPS paired specimens		
	concordant cases	discordant cases	*P* value
	*n* = 349	*n* = 91	
Gender (boy)	211	58	0.568
Age (IQR)^a^	0.6 (0.3–1.1)	1.0 (0.4–1.7)	0.002

^a^The median age is shown in years

## Discussion

Nucleic acid amplification techniques (NAATs) such as PCR and multiplex-PCR have been widely used to identify pathogens in infectious respiratory diseases [16, 17]. However, choosing the best sample type is a necessary prerequisite for optimal performance of these NAATs tests [4, 18]. Only a few reports have described different yield of sample types for microbiological detection in pediatric patients with lower respiratory tract infection [6, 7, 13]. In this study, we used multiplex-PCR to detect 9 virus and 2 atypical bacteria in 440 OPS-sputum specimen pairs from hospitalized children with lower respiratory tract infection (LRTI). We found almost perfect diagnosis agreement between these 2 specimens on nine viruses, but not for the atypical bacteria, *M. pneumoniae* or *C. pneumoniae*. In the cases with inconsistent results, more types of pathogens were identified from sputum than OPSs. Further analysis revealed that patients with consistent results were significantly younger than the children with inconsistent results.

Only a few studies have simultaneously tested the viral and bacterial yield of the same child between different specimen types [7, 13]. Lambert et al. measured eight types/subtypes of virus in 303 paired nose-throat swab/NP aspirate samples collected from children with respiratory symptoms. They found that NP aspirates improved sensitivity for the major viruses of childhood, except for influenza B, as its sensitivity was slightly higher in nose-throat swab specimens than NP aspirates (100% vs. 90%) [7]. Similar to our study, sputum testing was more sensitive for influenza B detection (91% vs. 89%). Huijskens explored respiratory pathogens in 92 adult CAP patients, and they discovered that the sensitivity of influenza A were similar in OPS and sputum, but influenza B from OPS was greater than sputum (100% vs. 33%) [19]. Based on these findings, OP or NP swab sample could reasonably replace NP/OP suction as an outpatient procedure for children to avoid missed diagnosis on influenza infection.

Thea et al. explored 10 bacteria and 18 viruses using the real-time PCR in 1692 children with severe pneumonia, the sputum specimens were found to increase the number of cases with any virus (4.1%) or bacteria (3.9%), but the overall added yield was marginal (1.3%) [13]. In the recent work, we also observed a 4% increase in sputum yields beyond OPS. Although the diagnostic detection of sputum specimen may not be sufficient to offset mild invasive procedure, sputum samples are still important in cases with certain pathogens are suspected, such as *B. pertussis* [20], *M. pneumoniae* [4, 13, 21–23]. In this study, the advantages of sputum as a specimen type for detection of *M. pneumoniae* and *C. pneumoniae* were concordant with previous findings. Raty et al. compared three sample types (sputum, NP aspirate and throat swab) from 33 young adult with pneumonia, and they discovered that the highest positive detection rate was from sputum [4]. So the sputum for PCR detection of *M. pneumoniae* was recommended by the British Thoracic Society Guidelines (2009) [21]. Cho et al. also found a superior sensitivity of sputum in the *M. pneumoniae* and *C. pneumoniae* detection among 217 patients with CAP [22]. This outstanding diagnostic sensitivity of sputum PCR may be attributed to the different nature of MP and CP compared with the other respiratory pathogens than we tested for. As a higher copy number of *M. pneumoniae* [23] and *C. pneumoniae* [24] organisms was detected in the pulmonary alveoli than on the epithelium of the upper respiratory tract. These data together with ours indicate that LRTI cases who are suspected to infected with atypical bacteria, the sputum specimen is superior to OPS for reliable detection of MP and CP by PCR.

In the present study, we found that patients with consistent results between the two types of specimens were significantly younger than those with inconsistent results (median age: 0.6 vs.1.0 year). Thea et al. also observed that when the analysis was restricted to children< 6 months, the added yield of sputum over OPS was slightly reduced [13]. Numerous studies have reported that most young children are at high risk of pneumonia associated with viral pathogens [25–27]. Because of the high rate of virus detection in both sample types observed in our work, younger patients were more likely to show consistent results. Furthermore, consideration should be given that the induced sputum requires a vacuum system, suction catheter, mucus trap and better trained personnel, whereas OPS requires only a swab. Therefore, in order to avoid invasive procedures for sputum suction in young children, OPS will be the preferred sample type for pathogen detection.

## Conclusions

In conclusion, multiplex-PCR assay for detection of 11 types of pathogens from sputum samples yielded results similar to OPS except for *M. pneumoniae* and *C. pneumoniae*. Specimen type is critical in molecular diagnosis, and the convenience of the sampling procedure OPS would be the preferred sample type for young children.

## Additional file

Additional file 1: List of target pathogens. (XLSX 13 kb)

## Abbreviations

ADV: adenovirus
CP: *Chlamydia pneumoniae*
HBoV: human bocavirus
HCoV: human coronavirus
HMPV: human metapneumovirus
HPIV: human parainfluenza virus
HRV: human rhinovirus
InfA: influenza A
InfB: Influenza B
MP: *Mycoplasma pneumoniae*
RSV: respiratory syncytial virus
RT: reverse transcription
